# Supplementary material for: Multi-Wavelength Excitable Multicolor Upconversion and Ratiometric Luminescence Thermometry of Yb3+/Er3+ Co-Doped NaYGeO4 Microcrystals
Source: Molecules. 2024 Oct 15;29(20):4887. doi: 10.3390/molecules29204887 (PMC11510309; doi:10.3390/molecules29204887)
Supplement: Supplementary file 1 [file molecules-29-04887-s001.zip › molecules-3233837-supplementary.pdf]

Supplementary Information

Multi-wavelength excitable multicolor upconversion and ratiometric luminescence thermometry of Yb^{3+}/Er^{3+} co-doped $NaYGeO_4$ microcrystals

Hui Zeng, Yangbo Wang *, Xiaoyi Zhang, Xiangbing Bu, Zongyi Liu and Huaiyong Li *

School of Materials Science and Engineering, Laboratory of Sensitive Materials and Devices Shandong Department of Education, Liaocheng University, Liaocheng 252059, China

*Correspondence: wangyangbo@lcu.edu.cn; lihuaiyong@lcu.edu.cn

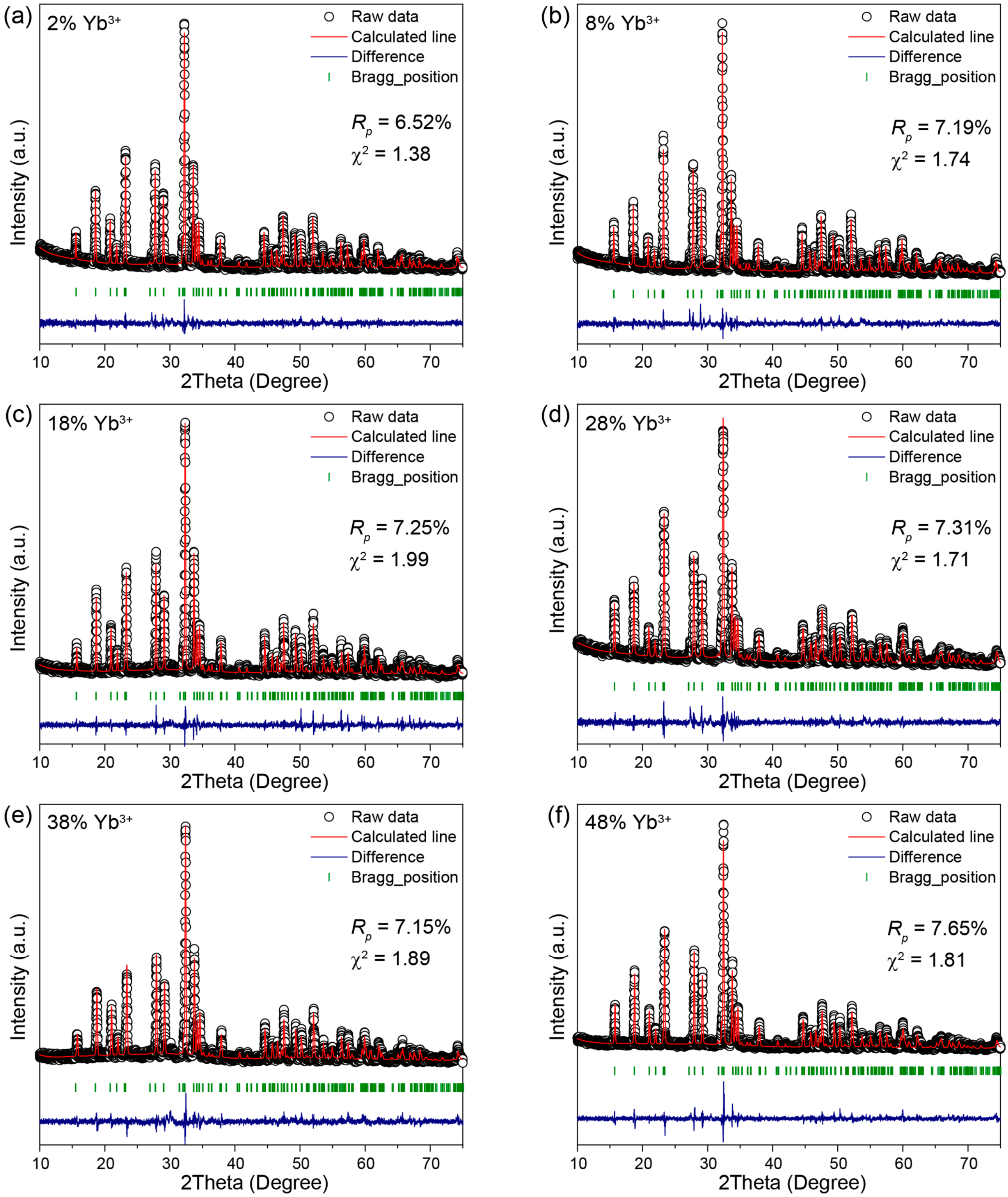

Figure S1. Rietveld refinement results of XRD patterns of $NaYGeO_4$:xYb^{3+}/2%Er^{3+} microcrystals, x = 2–48%.

Table S1 Rietveld refinement data of XRD patterns for $NaYGeO_4$:xYb^{3+}/2%Er^{3+} microcrystals, x = 2–48%.

Parameter	2%Yb^{3+}	8%Yb^{3+}	18%Yb^{3+}	28%Yb^{3+}	38%Yb^{3+}	48%Yb^{3+}
Space group	*Pnma*	*Pnma*	*Pnma*	*Pnma*	*Pnma*	*Pnma*
a (Å)	11.3810	11.3548	11.3530	11.3422	11.3364	11.3209
b (Å)	6.4273	6.4151	6.4115	6.4097	6.4008	6.3947
c (Å)	5.2601	5.2544	5.2500	5.2489	5.2380	5.2331
$\alpha=\beta=\gamma$ (°)	90	90	90	90	90	90
V (Å^3)	384.7716	382.7419	382.1462	381.5956	380.0799	378.8438
R_{wp} (%)	8.43	9.56	9.45	9.60	9.03	9.67
R_p (%)	6.52	7.19	7.25	7.31	7.15	7.65
χ^2	1.38	1.74	1.99	1.71	1.89	1.81

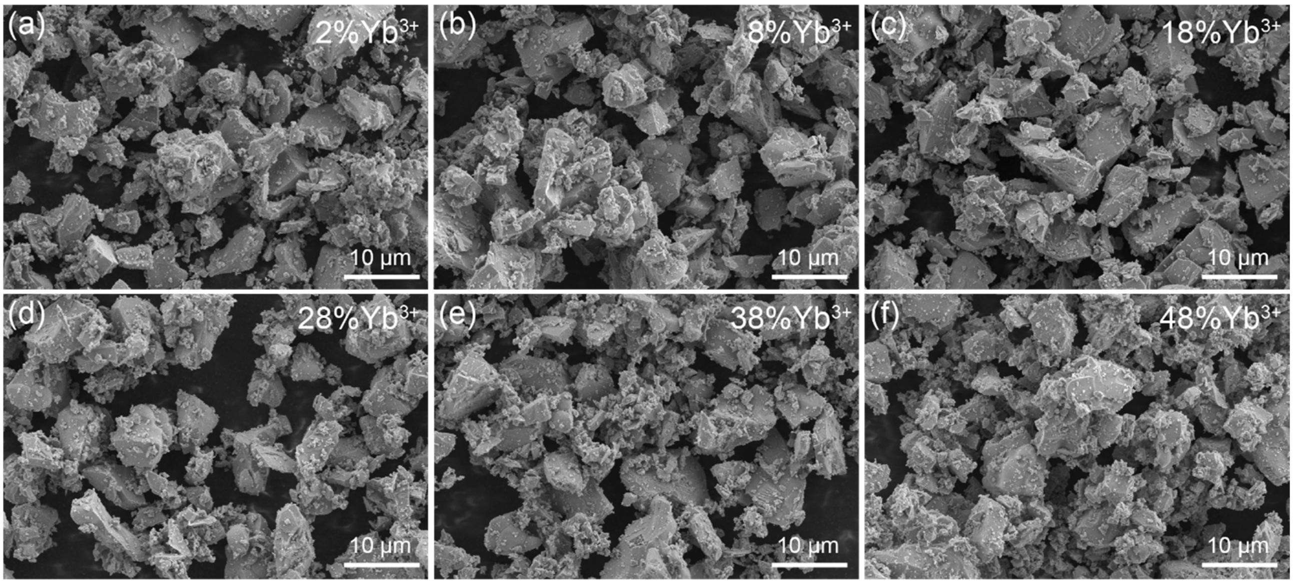

Figure S2. SEM images of $NaYGeO_4$:xYb^{3+}/2%Er^{3+} microcrystals, x = 2–48%.

Table S2 Detected atomic percentages by EDS spectra of $NaYGeO_4$:xYb^{3+}/2%Er^{3+} microcrystals, x = 2–48%.

Samples	Na (at%)	Y (at%)	Ge (at%)	O (at%)	Yb (at%)	Er (at%)
$NaYGeO_4$:2%Yb^{3+}/2%Er^{3+}	12.3	15.3	13.3	58.3	0.4	0.3
$NaYGeO_4$:8%Yb^{3+}/2%Er^{3+}	11.6	13.4	12.8	60.3	1.6	0.4
$NaYGeO_4$:18%Yb^{3+}/2%Er^{3+}	12.2	12.1	13.3	58.5	3.6	0.3
$NaYGeO_4$:28%Yb^{3+}/2%Er^{3+}	12.4	11.0	13.3	56.9	6.1	0.2
$NaYGeO_4$:38%Yb^{3+}/2%Er^{3+}	13.0	9.1	13.3	56.9	7.5	0.3
$NaYGeO_4$:48%Yb^{3+}/2%Er^{3+}	12.7	7.7	13.4	56.1	10.0	0.2

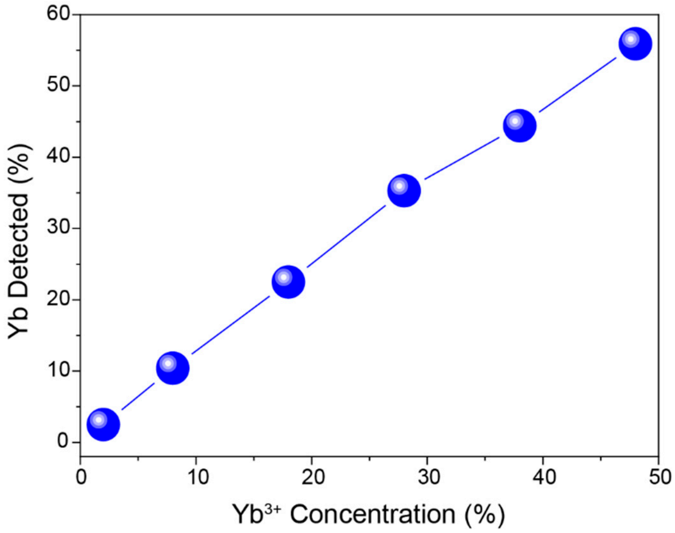

Figure S3. Variation of detected Yb concentration on Yb^{3+} doping concentration.

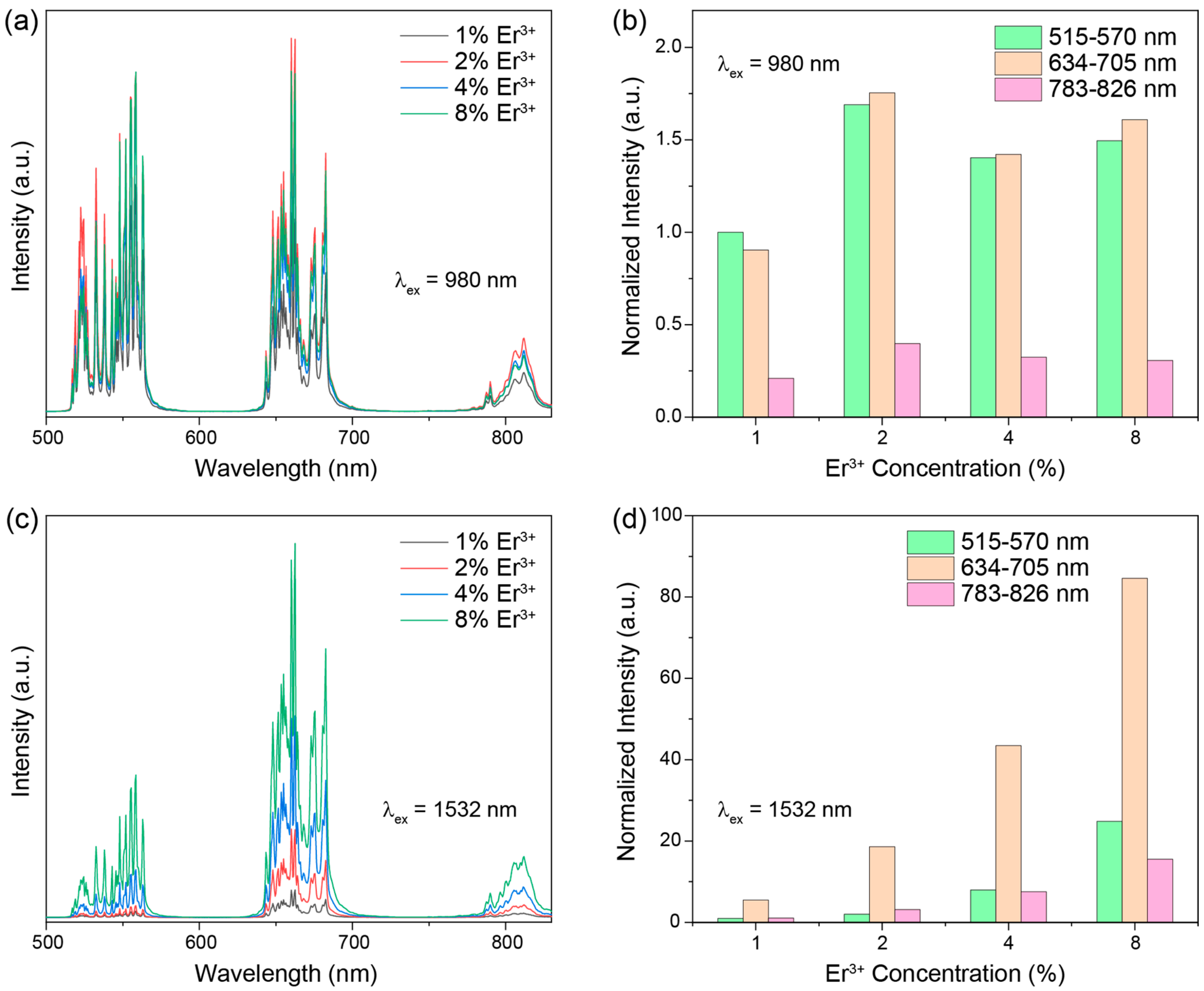

Figure S4. (a, c) Upconversion emission spectra and (b, d) corresponding integral intensity evolutions with increased Er^{3+} concentrations of $NaYGeO_4$:18%Yb^{3+}/yEr^{3+}, y = 1–8%, under (a, b) 980 and (c, d) 1532 nm laser excitation.

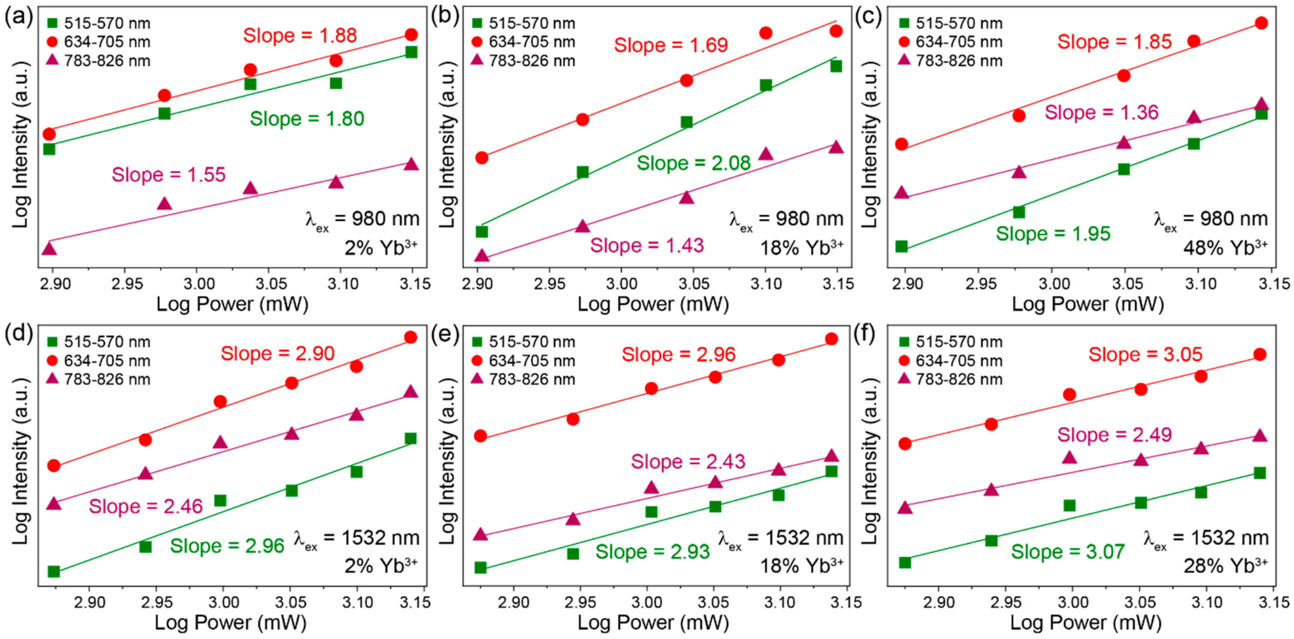

Figure S5. The double logarithmic plots of the integral intensities for 515–570, 634–705, and 783–826 nm upconversion emissions of $NaYGeO_4$:xYb^{3+}/2%Er^{3+} microcrystals: (a–c) 980 nm excitation, (d–f) 1532 nm excitation.

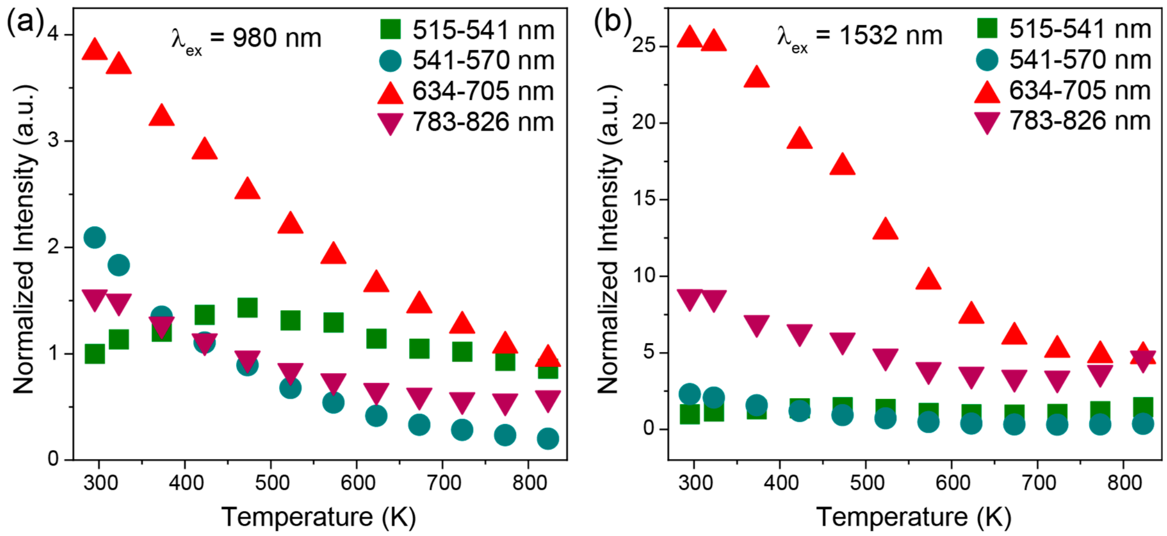

Figure S6. Temperature dependences (295–823 K) of integral intensities for 515–541, 541–570, 634–705, and 783–826 nm upconversion emissions of $NaYGeO_4$:18%Yb^{3+}/2%Er^{3+} microcrystals, under (a) 980 and (b) 1532 nm excitation.
